# TRIM24 Overexpression Is Common in Locally Advanced Head and Neck Squamous Cell Carcinoma and Correlates with Aggressive Malignant Phenotypes

**DOI:** 10.1371/journal.pone.0063887

**Published:** 2013-05-22

**Authors:** Zhibin Cui, Wei Cao, Jiang Li, Xiaomeng Song, Li Mao, Wantao Chen

**Affiliations:** 1 Department of Oral and Maxillofacial Surgery, Ninth People’s Hospital, Shanghai Jiao Tong University School of Medicine, Shanghai, China; 2 Shanghai Key Laboratory of Stomatology, Shanghai, China; 3 Department of Oral Pathology, Ninth People’s Hospital, Shanghai Jiao Tong University School of Medicine, Shanghai, China; 4 Department of Oncology and Diagnostic Sciences, University of Maryland School of Dentistry, Baltimore, Maryland, United States of America; University of Illinois, United States of America

## Abstract

Tripartite motif-containing 24 (TRIM24), a member of the transcriptional intermediary factor 1 family, functions as a co-regulator that positively or negatively modulates the transcriptional activities of several nuclear receptors. The aim of this study was to investigate TRIM24 expression and its clinical significance in head and neck squamous cell carcinoma. The expression levels of TRIM24 variants were examined in head and neck squamous cell carcinoma (HNSCC) samples and cell lines by real-time PCR and WB. The expression levels of TRIM24 measured in 91 locally advanced HNSCC tumors were measured by immunohistochemistry and correlated with clinical and pathological parameters. The functional role of TRIM24 in HNSCC was further investigated by silencing its expression in HNSCC cell lines. TRIM24 variants were up-regulated in 56 HNSCC samples (*P*<.001) and 9 HNSCC cell lines (*P*<.05). TRIM24 protein was overexpressed in 6 of 8 HNSCC cell lines and in 2 of 3 HNSCC samples. Furthermore, 54.95% (50/91) of HNSCC samples exhibited remarkably elevated expression of TRIM24 by immunohistochemistry. Univariate analysis revealed that high TRIM24 expression was associated with worse overall survival (*P* = .020). In multivariate analysis, TRIM24 expression was identified as an independent predictor of overall survival (*P* = .030), after adjusting for other clinicopathological parameters. Upon TRIM24 silencing, the proliferation of HNSCC cells was notably inhibited due to the induction of apoptosis. These results suggest that aberrant TRIM24 expression may play an important role in the development of HNSCC and is a promising prognostic indicator for patients with locally advanced HNSCC.

## Introduction

Head and neck squamous cell carcinoma (HNSCC) is one of the 10 most common malignancies in the world with more than 500 000 new cases are reported annually. It is widely recognized as a heterogeneous tumor type with various aggressive malignant phenotypes [1]. For those with locally advanced tumors, the 5-year survival rate is approximately 40%. It is well established that the development of HNSCC is a multi-step process in which the activation of oncogenes and inactivation of tumor suppressor genes, such as inactivation of tumor suppressor genes *p53* and *p16* and activation of oncogenes cyclin D1 (*CCND1*) and epidermal growth factor receptor (*EGFR*), play critical roles [2], [3], [4], [5]. Better understanding the molecular basis of HNSCC should further facilitate development of novel strategies to improve early diagnosis and treatment of the disease.

Tripartite Motif-Containing 24 (TRIM24) belongs to a subgroup of the large family of tripartite-motif (TRIM) proteins. All TRIM proteins contain several conserved domains: a RING finger, one or two zinc-binding motifs named B-boxes and a coiled-coil region [6]. It was reported that the three conserved motifs work together to degrade wild-type p53 protein by tagging it with ubiquitin [7], [8]. The inactivation of TRIM24 together with TRIM28 or TRIM33 in mice disturbs RA-signaling in hepatocytes, resulting in the development of hepatocellular carcinoma in a cell-autonomous manner [9], [10]. Additionally, in vitro studies have suggested that TRIM24 negatively regulates prostate cancer cell proliferation through binding to bromodomain containing 7, which represses androgen receptor transactivation activity [11]. Meanwhile, TRIM24 can bind chromatin and the estrogen receptor to activate estrogen-dependent genes associated with cell proliferation and tumor development in breast [12]. Furthermore, the aberrant expression of TRIM24 is significantly associated with poor prognosis patients with breast cancer [13].

In this study, we evaluated the expression profile of TRIM24 in patients with HNSCC and found that TRIM24 is frequently overexpressed. The up-regulation of TRIM24 was significantly associated with worse overall survival in patients with locally advanced HNSCC. Furthermore, our data indicate that TRIM24 plays a critical role in promoting growth and survival of HNSCC cells in vitro. To the best of our knowledge, our results provide the first evidence suggesting TRIM24 as an independent prognostic indicator for patients with locally advanced HNSCC.

## Materials and Methods

### Patients and Specimens

TRIM24 mRNAs were evaluated by real-time PCR in 56 pairs of HNSCC samples and adjacent normal tissues. The samples were obtained from patients with locally advanced HNSCC who were diagnosed at the Ninth People’s Hospital of the Shanghai Jiao Tong University School of Medicine from January 2007 to June 2009. All samples were collected post-surgery and immediately frozen in liquid nitrogen until total RNA was extracted. Another 91 independent specimens were obtained from patients with locally advanced HNSCC and examined for TRIM24 protein expression by immunohistochemical staining. All of the patients signed written informed consent in accordance with the institutional guidelines. Those patients were diagnosed at the Ninth People’s Hospital of the Shanghai Jiao Tong University School of Medicine from 1999 to 2004. The medical records of the patients were reviewed for inclusion and exclusion criteria. Exclusion criteria included patients with recurrent tumors; preoperative radiotherapy and/or chemotherapy; clinical stage I/II diseases; distant metastasis; and incomplete medical records. This cohort included 67 men (73.6%) and 24 women (26.4%) with a median follow-up of 102 months (interquartile range, 78-110 months). Detailed clinicopathological parameters are summarized in Table 1. The tumors from each patient were stained with hematoxylin and eosin (H&E), classified histologically, and staged according to the International Union Against Cancer TNM classification system (5th Edition). Each sample has more than 50% of malignant tissue.

**Table 1:** Clinicopathological parameters of 91 patients with HNSCC.

Characteristics	Patients	
	No.	%
**Age, years**		
≥60	61	67.0
<60	30	33.0
**Sex**		
Male	67	73.6
Female	24	26.4
**Smoking history**		
Smoker	43	47.3
Non-smoker	41	45
Missing	7	7.7
**Alcohol use**		
Drinker	62	68.1
Non-Drinker	22	24.2
Missing	7	7.7
**Tumor grade**		
I	71	78.1
II-III	20	21.9
**TNM stage**		
III	32	35.2
IVa	59	64.8
**Lymph node metastasis**		
pN0	56	61.5
pN1-pN2	35	38.5
**Disease site**		
Oral cavity	78	85.7
Oropharynx	13	14.3
**Histologic signs of severity**		
Present	51	56.0
Absent	37	40.7
Missing	3	3.3

### Real-Time PCR Analysis

Two TRIM24 isoforms [13] were analyzed using two pairs of primers to detect variants 1 and 2. A primer set, which amplifies both isoforms (referred as “core”), was used as a control. The expression levels of TRIM24 variants were examined in 9 HNSCC cell lines, 1 immortalized keratinocyte cell line and 1 primary normal keratinocyte culture, as well as in 56 HNSCC samples and the paired adjacent normal tissues by real-time PCR. All real-time PCR reactions were performed as previously described [14]. The primers used for TRIM24 variants were as follows: 5′-AATGGACCAGTTCTTCCTCCTCA-3′ and 5′-TGGCTTTATTGCTTGTCGTGG-3′ for total TRIM24; 5′-GTTTACCAAACCCTAGAATGCAGG-3′ and 5′-AAGTTTATCAAACGTGGAGGCG-3′ for TRIM24 variant 1; 5′-CAGCAACAGCAACCGCCT-3′ and 5′-TGAGGAGGAAGAACTGGTCCATT-3′ for TRIM24 variant 2; 5′-CCTGGCACCCAGCACAAT-3′ and 5′-GGGCCGGACTCGTCATACT-3′ for β-actin. Primers for the nuclear factors are as follows: 5′-AAGCCCGAGTGCTCTGAGA-3′ and 5′-TTCGTAGTGTATTTGCCCAGC-3′ for RARA; 5′-TCCGAAAAGCTCACCAGGAAA-3′ and 5′-GGCCAGTTCACTGAATTTGTCC-3′ for RARB; 5′-GGCTACCAAGTGCATCATCAA-3′ and 5′-TGTGCAGATACGCAGCATCAG-3′ for RARC; 5′-AGTCATCGGTCAGACACCCTT-3′ and 5′-GTGCAGCGTTATCTCCAACAG-3′ for AR. The ΔΔCt method was applied to calculate the TRIM24 expression levels in tumor tissues relative to the normal adjacent tissues. Here, ΔCt is the difference in the Ct values derived from the specific genes compared with β-actin. ΔCt T (Tumor) = Ct value (TRIM24 in tumor tissue) - Ct value (β-actin in tumor tissue), whereas ΔCt N (Normal) = Ct value (TRIM24 in normal tissue)-Ct value (β-actin in normal tissue). The relative expression levels of TRIM24 mRNA normalized to β-actin were represented as –ΔCt value here. Thus, higher ΔCt values correspond to lower relative expression levels of TRIM24 mRNA. The significance level was set at *P*<.01.

### Western Blot Analysis

Cells and tissue samples were lysed in radioimmunoprecipitation assay buffer (Sigma-Aldrich, St. Louis, MO). The lysates were separated by sodium dodecyl sulfate-polyacrylamide gel electrophoresis. Primary antibodies against TRIM24 (Proteintech, Chicago, IL) were used to evaluate protein levels, and GAPDH or β-actin antibody (Santa Cruz Biotechnology Inc., Santa Cruz, CA) was used to normalize the protein loading. Bands were detected using an IRDye 800-conjugated affinity-purified antimouse immunoglobulin M antibody (Rockland, Gilbertsville, PA). The membrane was then washed several times and scanned using the Odyssey infrared imaging system (LICOR, Lincoln, NE) in the 800-channel wavelength and analyzed with Odyssey software. Finally, bands were quantified using the Image J processing and analysis software.

### Immunohistochemical Staining

The avidin-biotin complex (ABC) staining technique was performed according to the manufacturer’s instructions for the Vectastain Elite ABC kit (Vector Laboratories, Burlingame, CA), as described previously [15]. Briefly, tissue sections were deparaffinized in xylene, rehydrated in graded ethanol, treated with Tris-ethylene diamine tetraacetic acid buffer or citrate buffer for antigen retrieval, and quenched in hydrogen peroxide. The tissue sections were then blocked with 2.5% normal serum, incubated overnight at 4°C with anti-TRIM24 antibody (1∶500 dilution; Proteintech, Chicago, IL) followed by incubating with biotinylated secondary antibody and ABC reagent. Diaminobenzidine was used as chromogen, and sections were counterstained with Mayer hematoxylin (Sigma-Aldrich, St. Louis, MO). The labeling index was defined semi-quantitatively as the intensity of staining (0, 1, 2, or 3) multiplied by the percentage of positive tumor cells (25, 50, or 75). A pathologist randomly examined 7 to 10 tumor areas in each section, and the final scores ranging from 0 to 300 were calculated. For TRIM24 staining, we divided the cases into 4 grades by scores. The cases scored from 0 to 25 were marked as negative (-); cases scored from 25 to 100 were marked as low grade (+); cases scored from 100 to 225 were marked as median grade (++); and cases scored from 225 to 300 were marked as high grade (+++). We also defined the negative (-) and low (+) grades cases as low TRIM24 expression whereas the median (++) and high (+++) grades cases as high TRIM24 expression.

### Cell Culture

The HNSCC cell lines WSU-HN4, HN6, HN12, HN13, and HN30 (kindly provided by the University of Maryland School of Dentistry) were cultured in Dulbecco’s modified Eagle medium (DMEM) (GIBCO-BRL, Grand Island, NY) supplemented with 10% heat-inactivated fetal bovine serum (FBS) (GIBCO-BRL) penicillin (100 unit/mL), and streptomycin (100 µg/mL) at 37°C in a humidified 5% CO_2_ atmosphere. CAL27, SCC4, SCC9, and SCC25 cells (purchased from the American Type Culture Collection, Manassas, VA) were cultured in DMEM/F12 medium (GIBCO-BRL) supplemented with 10% heat-inactivated FBS, penicillin (100 unit/mL), and streptomycin (100 µg/mL). Immortalized oral epithelial cells infected with human papillomavirus type 16-E6/E7 (HPV16E6/E7) were cultured in a defined keratinocyte serum-free medium (GIBCO-BRL) [16]. Normal human oral mucosa were harvested from the area adjacent to the area of tooth extraction at Oral and Maxillofacial Surgery outpatient clinic. The tissue was incubated with dispase overnight to separate the surface epithelium from the underlying fibrous connective tissue. The epithelium was then trypsinized to prepare a single cell suspension. Primary normal human oral mucosa cells were cultured in the keratinocyte serum-free medium (GIBCO-BRL) as described in the previous study [17].

### Small Interfering RNA Transfection

Small interfering RNAs (siRNAs) targeting the two splice variants of TRIM24 and FAM-labeled negative control siRNA were purchased from Shanghai Genepharma Company, Ltd. (Shanghai, China). The siRNA sequences used were 5′-AAGCAGGTGGAACAGGATATTAAAGTTGC-3′ (siRNA for TRIM24) and 5′-ACGUGACACGUUCGGAGAATT-3′ (negative control siRNA). Sequences of TRIM24-siRNA1 and TRIM24-siRNA2 in Figure S1 were 5′- AAAGCCAGGCTCATTTCATGG-3′ and 5′-AAAGTAATGCTGTACTGCTGC-3′. In vitro transient transfection was performed using Lipofectamine 2000 (Invitrogen, Carlsbad, CA) as described previously [18].

### Cell Proliferation Assay

Cell-proliferation assay was performed to analyze the proliferation potential of WSU-HN6 and SCC25 cells that were transiently transfected TRIM24 siRNA or negative control siRNA. The cells were harvested and plated in 96-well plates at 1×10^3^ cells per well and maintained at 37°C in a humidified incubator. At the indicated time points, 10 µL of CCK-8 solution was added to the wells and incubated for 1 hour, and the absorbance at 450 nm was measured to calculate the number of viable cells in each well. Measurements were performed in triplicate, and the mean (standard deviation) optical density was reported.

### Colony Formation Assay

Briefly, TRIM24-siRNA-transfected and control-siRNA-transfected WSU-HN6 cells (1×10^3^ cells per plate) and SCC25 (2×10^3^ cells per plate) cells were seeded in a 10 mm plate and cultured in complete medium for 1 week. Cell colonies were then visualized by 0.25% crystal violet. After washing out the dye, colonies containing >50 cells were counted.

### Cell Apoptosis Assay

Apoptosis was assessed using Annexin V-PI staining 48 hours after transfection. Then, these cells were quantified by flow cytometry using the Annexin V-FITC Apoptosis Detection Kit (BD Biosciences, San Jose, CA, USA) according to the manufacturer’s protocols. Briefly, floating cells and trypsinized adherent cells were pooled and suspended in 100 µl of Annexin V- FITC Binding Buffer. Then, 5 µl of Annexin V-FITC and 5 µl of PI were added, and the cells were incubated for 15 minutes at 25°C. The cells were then resuspended in 400 µl of the Annexin V-FITC binding buffer and analyzed immediately by flow cytometry. Then, 10 000 events were scored by FACS Calibur and analyzed using Cell Quest software.

### Statistical Analysis

Real-time PCR and in vitro statistical analyses were performed in SPSS (standard version 13.0; SPSS Inc., Chicago, IL). The real-time PCR analysis results were evaluated using the Mann-Whitney test for two independent groups. The results of the cell proliferation assay, colony formation assay, and in vitro apoptosis assay were evaluated using Student’s *t*-tests. The log-rank test was used to analyze univariate associations between TRIM24 expression pattern and overall survival. Then, all potential prognostic factors with *P* values <.05 from the univariate analysis were incorporated into multivariate analyses. Hazard ratios with corresponding 95% confidence intervals (CIs) and *P* values were reported. All tests were two-sided, and *P* values <.05 were considered statistically significant.

## Results

### Overexpression of TRIM24 in HNSCC

**Figure 1:**
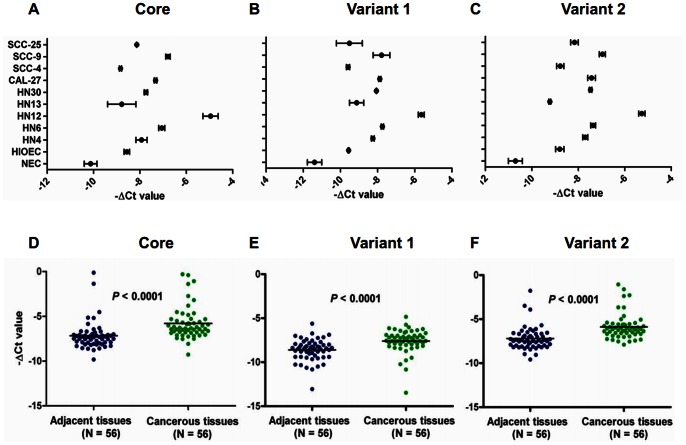
TRIM24 mRNA expression in HNSCC cell lines and primary HNSCC tissues. (A-C) core (control) and two variants of TRIM24 mRNA expressed in HNSCC cell lines compared to primary keratinocytes. (D-F) Core and two variants of TRIM24 mRNA were expressed in 56 primary HNSCC tissues and their paired adjacent normal tissues.

**Figure 2:**
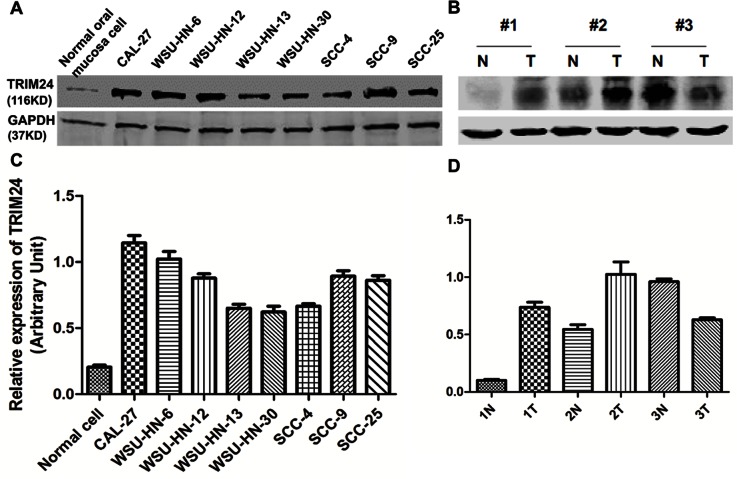
TRIM24 protein expression in HNSCC cell lines and primary HNSCC tissues. (A) TRIM24 protein detected in 8 HNSCC cell lines. (B) Protein levels of TRIM24 in 3 paired HNSCC tissues and the paired adjacent normal tissues. (C) and (D) protein expression levels of TRIM24 quantified using Image J processing and analysis software.

Both core and two variants of TRIM24 mRNA were expressed higher in the 9 HNSCC cell lines and the immortalized cell line than in the primary normal keratinocytes (Fig. 1A–C). Similarly, TRIM24 mRNA levels were higher in HNSCC tissues compared to adjacent normal tissues in the 56 HNSCC patients (Fig. 1D–F). On Western blots, TRIM24 protein is highly expressed in all HNSCC cell lines comparing to the normal oral mucosa cells. While in the HNSCC cell lines, HN30 and HN13 and SCC4 was expressed lowly than the other HNSCC cell lines (Figure 2 A). In 3 paired HNSCC tissues and their adjacent normal tissues, 2 of the 3 showed higher TRIM24 levels in the tumors than in the adjacent normal tissues (Fig. 2).

### TRIM24 Protein Overexpression Correlates with Poor Prognosis in HNSCC Patients

**Figure 3:**
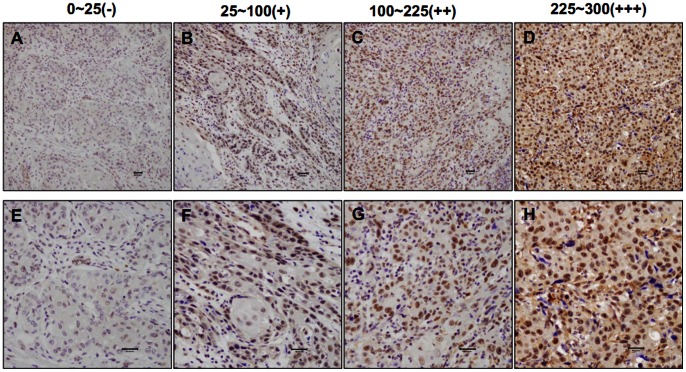
Immunohistochemical staining of TRIM24 expression in 91 tumors from HNSCC patients. (A-D) Different patterns of TRIM24 expression in HNSCC tissues (200X). (A) Example of scores 0-25 (-); (B) Scores 25-100 (+); (C) Scores 100-225 (++); and (D) Scores 225-300 (+++). (*E-H)* Different patterns of TRIM24 expression in HNSCC tissue (400X).

**Figure 4:**
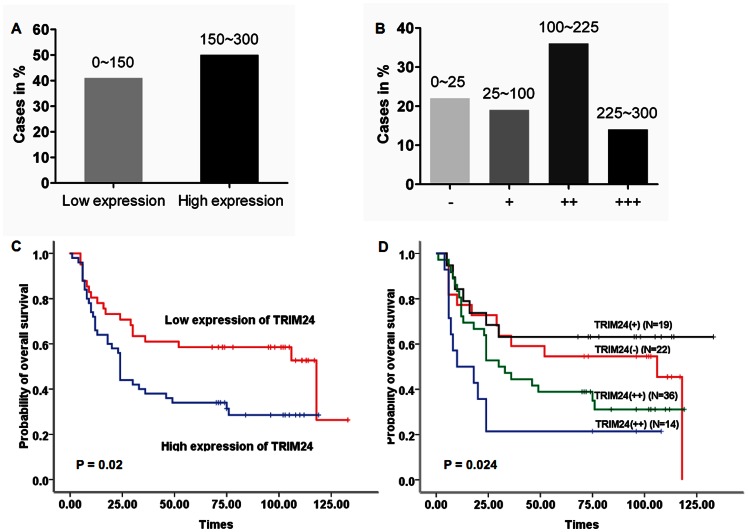
Analysis of TRIM24 expression with its clinical prognosis. (A and B) Results of TRIM24 expression status were showed in the 91 HNSCC tissues; (C and D) Kaplan-Meier survival curves for overall survivals based on TRIM24 staining patterns.

We investigated TRIM24 protein expression pattern using immunohistochemical staining in 91 tumor samples from patients with locally advanced HNSCC and analyzed the correlations between TRIM24 expression and clinical outcome using Kaplan-Meier survival analysis. TRIM24 staining was mainly located in the nuclei of the tumor cells, as shown in Figure 3. TRIM24 was expressed at high level in 54.9% (50/91) the tumors (Fig. 4A). Notably, patients whose primary tumors exhibited high levels of TRIM24 (the mean labeling index of 150 was used as a cutoff value) had significantly poorer overall survival (*P* = .020) (Fig. 4C). To further analyze the correlation, we classified the expression level of TRIM24 into four subgroups and performed further correlation analyses. Twenty-two patients (24.2%) were negative for TRIM24 (-), nineteen (20.9%) patients were classified as having low TRIM24 expression (+), thirty-six (39.6%) were classified as median (++), and fourteen (15.3%) were classified as high (+++) (Fig. 4B). A expression level dependent relationship with the overall survivals was observed (*P* = .024) (Fig. 4D).

### Univariate and Multivariate Survival Analysis

Univariate and multivariate survival analyses were performed to further evaluate whether TRIM24 expression is an independent prognostic indicator in locally advanced HNSCC patients. In the univariate Cox proportional analysis, TRIM24 expression (hazard ratio, 1.910; 95% CI, 1.088-3.352; *P* = .024), tumor grade (hazard ratio, 1.825; 95% CI, 1.060-3.143; *P* = .030), lymph node metastasis (hazard ratio, 2.269; 95% CI, 1.390-3.703; *P* = .001) and histological signs of severity (including vascular embolization, perineural invasion, and diffuse invasion. Hazard ratio, 2.636; 95% CI, 1.538-4.520; *P*<.001) were significantly correlated with the overall survival of the patient population (Table 2). In the multivariate analysis, after adjusting for tumor grade and lymph node metastasis, only TRIM24 expression (hazard ratio, 1.910; 95% CI, 1.064-3.428; *P* = .030) and lymph node metastasis (hazard ratio, 2.837; 95% CI, 1.492-5.396; *P* = .018) were identified as independent factors contributing to the worse overall survival (Table 3).

**Table 2:** Univariate Cox Proportional Hazards Regression Models for Estimating Overall Survival.

Characteristics	HR	95%CI	*P*
Age (≥60 vs <60)	1.091	0.672 to 1.773	.724
Sex (male vs female)	0.777	0.443 to 1.365	.381
Smoking history(smoker vs nonsmoker)	0.896	0.548 to 1.465	.662
Alcohol history(drinker vs nondrinker)	0.925	0.539 to 1.585	.776
Tumor grade (I vs II-III)	1.825	1.060 to 3.143	.030
TNM stage (III vs IVa)	1.538	0.909 to 3.703	.109
Lymph node metastasis (pn0vs pn1-pn2)	2.269	1.390 to 3.703	.001
TRIM24 expression (high vs low)	1.910	1.088 to 3.352	.024
Disease site (oral cavity vsoropharynx)	1.397	0.712 to 2.742	.331
Histologic signs of severity(presentvs absent)	2.636	1.538 to 4.520	<.001

**Table 3:** Multivariate Cox Proportional Hazards Regression Models for Estimating Overall Survival.

Characteristics	HR	95%CI	P
Tumor grade (I vs II-III)	1.540	0.815 to 2.912	.184
TRIM24 expression (High vs Low)	1.910	1.064 to 3.428	.030
Lymph node metastasis(pN0 vs pN1-pN2)	1.418	0.801 to 2.510	.231
Histologic signs of severity(present vs absent)	2.837	1.492 to 5.396	.001

### TRIM24 Silencing Inhibits Proliferation and Induces Apoptosis in HNSCC Cell Lines

**Figure 5:**
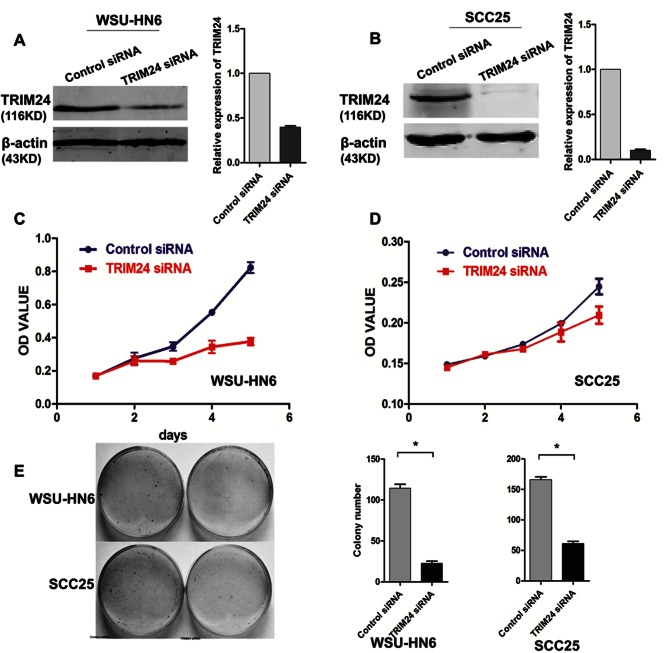
Down-regulation of TRIM24 in HNSCC cell lines. (A and B) Down-regulation effects of TRIM24-siRNA in WSU-HN6 and SCC25 cell lines. The right panel shows the quantified image; (C and D) Growth of WSU-HN6 and SCC25 cells with TRIM24 down-regulation using CCK-8; (E) Colony formation assay of WSU-HN6 and SCC25 cells with and without TRIM24 down-regulation.

**Figure 6:**
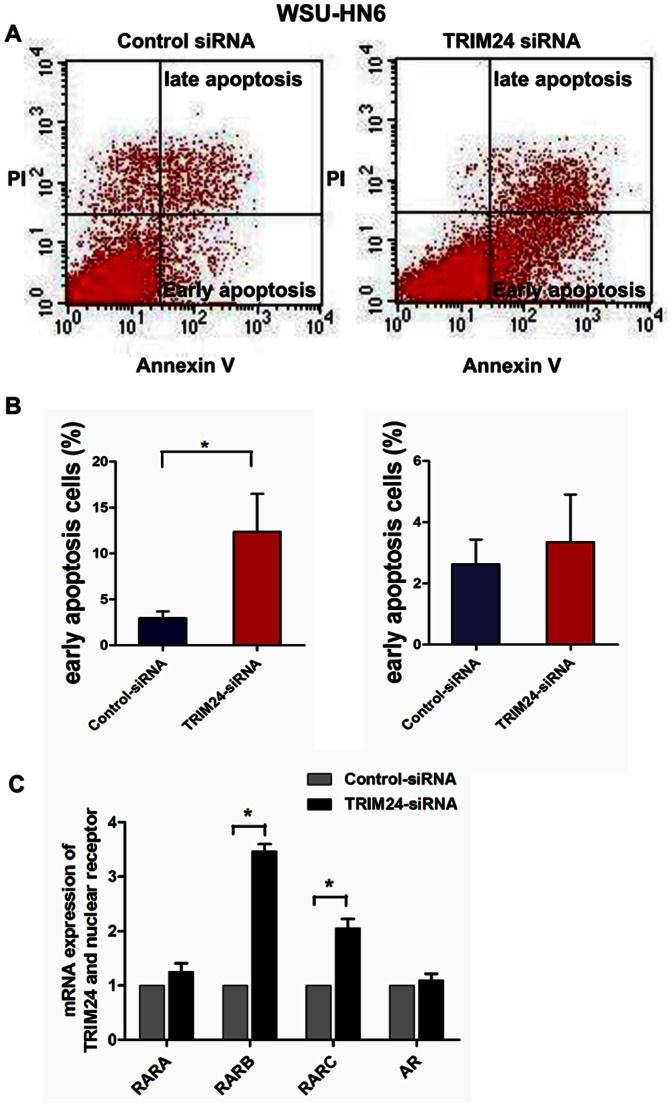
Apoptosis analysis of WSU-HN6 with TRIM24 down-regulation. (A) Annexin V-FITC-propidium iodide flow cytometry of WSU-HN6 cells with and without TRIM24 down-regulation; (B) Shows their quantified image; (C) mRNA expression of nuclear receptors in WSU-HN6 cells with TRIM24-siRNA and its control-siRNA.

To further investigate the role of TRIM24 in HNSCC progression, we down-related TRIM24 expression using siRNA in two selected HNSCC cell lines with high endogenous TRIM24 (SCC25 and WSU-HN6). The siRNA down-regulated TRIM24 levels in both cell lines (Fig. 5A and B). The down-regulation significantly inhibited growth of both WSU-HN6 (Day 5, *P*<.001) (Fig.5C) and SCC 25 (Day 5, *P*<.05) cells (Fig. 5D). To eliminate the side effects that may be caused by single siRNA, we redesigned another two siRNA and transfected into the WSU-HN6 cell line to detect the cell growth using CCK-8. Results showed that both the two siRNA of TRIM24 inhibited cell growth of WSU-HN6 cells (figure S1 A and B). TRIM24 down-regulation also significantly inhibited colony formation of WSU-HN6 (*P*<.01) and SCC25 (*P*<.01) cells (Fig. 5E). To determine whether the growth inhibition was due to the induction of apoptosis, we measured the apoptosis of the cells using Annexin V-FITC/PI double staining. The number of early apoptotic cells was significantly increased in WHU-HN6 cells transfected with TRIM24 siRNA at 48 hours compared with control siRNA-transfected cells (*P*<.05) (Fig. 6 A and B).

### TRIM24 Silencing Induces the Expression of Nuclear Receptors

Previous studies showed that TRIM24 regulates transcriptional activities of various nuclear receptors including RAR and AR. We then performed real-time PCR to test the expression profiles of RAR and AR in WSU-HN6 cell line treated with siRNA transfection. Finally we found that the down regulation of TRIM24 induces high expression of RARB (*P*<.0001) and RARC (*P* = .003), while there is no such correlation between TRIM24 and RARA (*P* = .17) and AR (*P* = .49) (Figure 6 C).

## Discussion

In this study, we found that TRIM24, also called TIF-1, was frequently up-regulated at both mRNA and protein levels in HNSCC, in cell lines and primary tumors. As previous studies showed that TRIM24 expression is up-regulated in breast cancer, myelodysplastic syndrome-related acute myeloid leukemia and sporadic colorectal cancers [13], [19], [20], our results support that aberrant expression of TRIM24 is common in multiple tumor types and may contribute to initiation and/or progression of these tumors. Using array CGH analysis, gains at the TRIM24 locus (7p34) were detected in approximately 10% of the breast cancers suggesting that genomic alterations may partly account for the TRIM24 overexpression in breast cancer [13]. Whether the genetic or the epigenetic factors contribute to the increased expression of TRIM24 in HNSCC remains to be determined in future studies. Earlier studies suggest that the increased levels of TRIM24 mRNA might not directly link to the increased protein levels due to other factors such as post-transcriptional regulation, epigenetic modifications or microRNA regulation [21], [22]. In this study, we measured both mRNA and protein levels of TRIM24 and found that TRIM24 expression was increased in the HNSCC cell lines and primary tumor tissues at both mRNA and protein levels.

An important goal of studying molecular abnormalities in cancers is to translate the knowledge into clinical applications [23]. To develop clinically useful biomarkers, we need to take steps to validate these markers. In this study, we applied immunohistochemical staining to determine TRIM24 expression status in an independent cohort of patients with HNSCC to validate findings obtained from our first set of patients. High expression of TRIM24 in the locally advanced HNSCC indicates it is common event in the tumor type. TRIM24 staining was found predominantly in nuclei, suggesting it may function as a transcriptional regulator in cancer cells as suggested in a previous study [13]. The finding that the aberrant expression of TRIM24 correlates with the poor survival of patients with HNSCC is significant because it suggests TRIM24 may play an important biological role in the progression of HNSCC and be used as a biomarker to predict clinical outcome of patients with HNSCC. Together with other biomarkers, TRIM24 status may help the implementation of optimized therapeutic strategies for HNSCC [24], [25].

A recent functional study, Allton et al. showed that TRIM24 (also called bonus in Drosophila) can negatively regulate p53 and that loss of TRIM24 can cause p53-dependent apoptosis in human breast cancer cells [7]. TRIM24 regulates the p53 degradation as a E3-ubiquitin ligase, while there are quantity of negative regulators (including other ubiquitin E3-ligases, i.e. MDM2) that could play the role in degrading the p53. During our study, we down-regulated the expression of TRIM24 alone and did not find the up-regulation of P53. Chambon et al., analyzed the tissue microarray of breast cancer samples and did not find correlations between TRIM24 expression and p53 status. The author discussed p53 E3-ubiquitin ligase activity by TRIM24 may be clearly tested in the liver-specific environment where p53 response to environmental stress like ultraviolet irradiation [13]. Meanwhile, Allton et al. validated the TRIM24 could bind to the p53 thus leads to the p53 degradation, while depletion of TRIM24 did not lead to an up-regulation of p53 (p>.05) in their supplementary data. According to the combination of the reported and our tests, we deemed that in HNSCC TRIM24 may degrade the p53 protein as a E3-ubiquitin ligase but may not be a drive factor resposible for p53 degradation and not directly influence the p53 protein stability. TRIM24 related p53 issue need far more research not only in HNSCC but also in most solid tumors.

Other studies showed that TRIM24 is overexpressed in acute promyelocytic leukemia and papillary thyroid carcinomas, respectively, and is a chromosomal translocation target of Braf (T18) and Ret oncogenes [18], [26], [27]. These results support the role of TRIM24 as an oncogene. However, using transgenetic animals, studies showed that TRIM24 may play a role in suppressing liver tumorigenesis [9], [10]. Such effect is possible because TRIM24 contains a nuclear receptor interaction domain (NRID) which can interact with many nuclear receptors such as retinoic acid receptor-alpha (RARα), retinoic acid receptor-X (RXR), vitamin D3 (VDR), estrogen (ER) and progesterone (PR) receptors [28]. These interactions make TRIM24 a co-regulator of various functions *via* trans-activation processes [29]. Thus, the roles of TRIM24 are complex and different in tumorigenesis of various organ systems. Nevertheless, our data support an oncogenic role of TRIM24 in HNSCC because not only the correlation between higher expression of TRIM24 and poorer clinical outcomes but also TRIM24 can promote growth and preventing apoptosis in HNSCC cells. Further studies will be needed to determine the mechanisms of TRIM24 in promoting head and neck tumorigenesis.

## Supporting Information

Figure S1 **Two TRIM24-siRNAs were used to eliminate the side effect in WSU-HN6 cells.** (A) Knockdown effects of TRIM24-siRNAs in WSU-HN6 cells. (B) Growth curve of WSU-HN6 with TRIM24-siRNAs and control-siRNA.
(TIF)
