# Supplementary figures and images for: TRIM24 Overexpression Is Common in Locally Advanced Head and Neck Squamous Cell Carcinoma and Correlates with Aggressive Malignant Phenotypes
Source: PLoS One. 2013 May 22;8(5):e63887. doi: 10.1371/journal.pone.0063887 (PMC3661592; doi:10.1371/journal.pone.0063887)

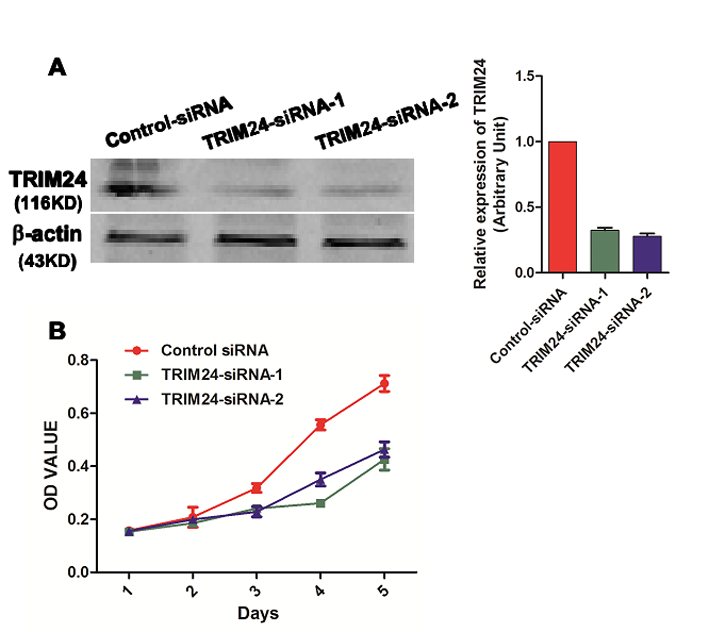

Supplement: Figure S1 — Two TRIM24-siRNAs were used to eliminate the side effect in WSU-HN6 cells. (A) Knockdown effects of TRIM24-siRNAs in WSU-HN6 cells. (B) Growth curve of WSU-HN6 with TRIM24-siRNAs and control-siRNA. (TIF) [file pone.0063887.s001.tif]
